# The Relationship Between Global and Domain‐Specific Evaluations of Life Satisfaction: A Feedback Loop Theory

**DOI:** 10.1111/jopy.70039

**Published:** 2025-12-12

**Authors:** Gabriele Prati

**Affiliations:** ^1^ Department of Psychology University of Bologna Cesena Italy

**Keywords:** bottom‐up, domain satisfaction, feedback loop, life satisfaction, top‐down

## Abstract

**Objective:**

Previous studies on the relationship between global (top‐down) and domain‐specific (bottom‐up) evaluations of life satisfaction have revealed mixed findings. The current study investigated the reciprocal relationship between top‐down and bottom‐up processes using two analytic methods to properly account for time‐varying predictors, confounding variables, and stable individual differences. Specifically, hierarchical Bayesian continuous‐time dynamic modeling and marginal structural models were employed.

**Method:**

Data from the Swiss Household Panel study (*N* = 25,181)—a nationally representative, longitudinal survey conducted in Switzerland—was used. Global life satisfaction was measured using the Satisfaction With Life Scale. Satisfaction with five life domains (i.e., health, income, personal relationships, free time, and job) was also measured.

**Results:**

A series of marginal structural models revealed that subsequent global life satisfaction was predicted by each domain‐specific evaluation, and prior global life satisfaction also predicted each domain‐specific evaluation. Continuous‐time dynamic modeling revealed that an independent shock of magnitude 1.00 to the top‐down process predicted future increases in the bottom‐up process, and vice versa.

**Conclusions:**

Taken together, the findings of this study can be represented by a closed‐loop feedback model, which illustrates predictive effects between top‐down and bottom‐up processes.

## 1 Introduction

Life satisfaction is a global evaluation of one's life as a whole and constitutes a major component of subjective well‐being (Busseri 2018; Diener et al. 1999). Life satisfaction is associated with a broad range of positive outcomes, including improved outcomes in the workplace (e.g., Oishi and Koo 2008; Walsh et al. 2018), better physical health indicators and lower mortality (e.g., Kim et al. 2021; Oishi and Koo 2008), marital adjustment (e.g., Be et al. 2013; Oishi and Koo 2008), higher levels of income (e.g., De Neve and Oswald 2012; Oishi and Koo 2008; Prati 2024a), reduced prejudice (Bazán‐Monasterio et al. 2021; Prati 2024b), and health behaviors (e.g., Kim et al. 2021; Oishi and Koo 2008). Over the last two decades, numerous studies have investigated life satisfaction (Diener et al. 2018; Tanwar and Khindri 2024).

Traditionally, two theoretical approaches have been proposed for explaining the evaluation of life satisfaction, differing in their causal assumptions: the ‘top‐down’ and the ‘bottom‐up’ perspectives (Busseri and Mise 2020; Diener 1984; Headey and Wearing 1992; Lance et al. 1989). According to the bottom‐up perspective, a global evaluation of one's life as a whole is a product of a range of domain‐specific satisfactions (Andrews and Withey 1976; Campbell et al. 1976). Accordingly, individuals' evaluation of global life satisfaction is a function of their experience of satisfaction in various life domains. From this perspective, therefore, the evaluations of various life domains (e.g., work, family, relationships, leisure, finances, health) are expected to make an independent contribution to the global evaluation of one's life (Heller et al. 2004). In contrast, the top‐down perspective assumes that global life evaluations influence domain‐specific evaluations (Diener 1984). In other words, “satisfaction with the domains may result from rather than cause global life satisfaction” (Diener 1984, 565). From this perspective, individuals who are more (vs. less) satisfied with their lives overall will be more likely to perceive greater (vs. lesser) satisfaction within various domains of their lives. While the bottom‐up perspective is an atomistic model subject to external contingencies, the ‘top‐down’ perspective is a dispositional explanation, which posits the notion of a global propensity to perceive, experience, and react to events (i.e., higher‐order or superordinate processes).

In the present work, the theoretical possibility that top‐down and bottom‐up perspectives might co‐exist within an individual has been considered. Moreover, it seems possible to hypothesize that the top‐down and the bottom‐up perspectives do not operate in isolation but rather exist in a reciprocal relationship, which is referred to as feedback loop theory (e.g., MacKay 1965; Miller et al. 1960; Wiener 1948). According to this theory, the relation between top‐down and bottom‐up processes may be represented by the closed‐loop feedback model. Specifically, this mutual process illustrates how the top‐down process influences the bottom‐up process, which, in turn, feeds back into the top‐down process. This third perspective on subjective well‐being posits that one's propensity to perceive life in a positive or negative light may influence domain‐specific evaluations via the situations individuals create for themselves (Diener 1984) and, in turn, different perceptions of situational characteristics are likely to affect individuals' evaluation of global life satisfaction (Andrews and Withey 1976; Campbell et al. 1976).

### 1.1 Previous Research on the Top‐Down/Bottom‐Up Controversy

In life satisfaction research, the top‐down/bottom‐up controversy has been discussed intensely (e.g., Busseri and Mise 2020; Diener 1984; Headey et al. 1991; Malvaso and Kang 2022; Scherpenzeel and Saris 1996). In one of the first attempts to empirically assess the direction between satisfaction in various life domains and global life satisfaction, Lance et al. (1989) found support for both models, and the directions of the relationships between life domains and global satisfaction varied across life domains. In this study, the authors asked a sample of the faculty of the University of Georgia to complete a questionnaire. They found that the relationships between global and life facet satisfaction varied across life domains. However, this study employed a cross‐sectional design, which does not allow the estimation of effects over time. Support for both the bottom‐up and top‐down models of life satisfaction was also found in subsequent research (Heller et al. 2004; Malvaso and Kang 2022). Heller et al. (2004) conducted a meta‐analysis of studies investigating top‐down and bottom‐up processes. They found that neuroticism, extraversion, agreeableness, and conscientiousness were linked to both various domain satisfactions and global life satisfaction, and that domain satisfactions were related to global life satisfaction. Malvaso and Kang (2022) used data from Wave 15 of the British Household Panel Study. Using multiple regression analysis, they found that personality is related to overall life satisfaction and that life domains predict overall life satisfaction. However, Heller et al. (2004) limited their searches to cross‐sectional studies and Malvaso and Kang (2022) used cross‐sectional data. Finally, support for both the top‐down and bottom‐up theories was provided in a diary study of 76 fully employed, married adults in the Iowa City area (Heller et al. 2006). Using multilevel regression, the authors found lagged effects that ran from domain satisfactions to global life satisfaction and vice versa. While this study has the merit of assessing within‐person associations, the sample was small and homogeneous. Moreover, because the study focused on day‐to‐day influences, it does not permit estimation of long‐term effects.

Headey et al. (1991) found more support for top‐down theory than bottom‐up theory, but overall, a complicated picture emerged. Headey et al. (1991) used data from four waves of the Australian Quality of Life panel survey and employed a cross‐lagged panel model (CLPM) to test the effects. While the CLPM may overcome the problems associated with the use of cross‐sectional research, it may lead to biased conclusions regarding the statistical significance and sign of the estimates (Hamaker et al. 2015). Greater support for top‐down theory was confirmed in subsequent research (Leonardi et al. 2005). Leonardi et al. (2005) analyzed data from 297 respondents in a European longitudinal survey. They used two measurement occasions and stepwise regression (method forward) to test predictions derived from top‐down and bottom‐up theories. However, two waves of measurement are considered insufficient for investigating effects over time (Ployhart and MacKenzie 2015). Finally, there is evidence indicating greater support for the bottom‐up perspective than for the top‐down model (Busseri and Mise 2020). Busseri and Mise (2020) used data from Wave 2 and Wave 3 of the Midlife in the United States (MIDUS) study. They used multiple regression to test aspects of the bottom‐up and top‐down perspectives simultaneously. They found that life satisfaction is more a function of situational factors than of dispositional factors. While the findings are noteworthy, the study's conclusions are constrained by its two‐wave design.

Although predictions derived from top‐down and bottom‐up perspectives may be valid, the conclusions drawn from these studies remain inconsistent. Moreover, these findings are derived from suboptimal research designs or analytical strategies for causal inference. Causal inference in observational research is notoriously challenging (Hernán and Robins 2020). One approach to addressing causal inference in observational research involves focusing on within‐person dynamics (Hamaker 2023) after separating within‐person and between‐person variability (Curran and Bauer 2010; Hamaker 2023). Conflating within‐person and between‐person variability may have contributed to inconsistencies in previous findings. From a conceptual and empirical point of view, within‐person dynamics are different from between‐person dynamics (e.g., Curran and Bauer 2010; Zhang and Wang 2014). Within‐person dynamics refer to an effect within a given individual (i.e., intraindividual processes), while between‐person dynamics denote effects across a set of individuals (i.e., interindividual processes). For instance, in the relationship between satisfaction with a life domain and overall life satisfaction, the between‐person relationship reflects the extent to which individuals who have more satisfaction with a life domain differ in overall life satisfaction from people who have less satisfaction with a life domain. In contrast, the within‐person relation reflects the extent to which individuals experience higher overall life satisfaction after experiencing high satisfaction with a life domain than when they do not. The investigation of the within‐person relations that are disaggregated from the between‐person relations is crucial for understanding effects over time within the same person, as it controls for the presence of stable individual differences (e.g., genetic influences on life satisfaction). Moreover, the conflation of between‐person and within‐person sources of variability (as in standard regression or CLPM) is likely to result in biased parameters and misleading inference (e.g., Curran and Bauer 2010; Hamaker 2023; Zhang and Wang 2014). Therefore, the use of a statistical technique that is able to separate both processes is important for causal inference (Hamaker 2023). Finally, Heller et al. (2006) highlighted the importance of investigating intra‐individual variability in life satisfaction. Indeed, a substantial amount of intra‐individual variation in life satisfaction has been found in their study. Using a random‐intercept cross‐lagged panel model (RICLPM) that disentangled between‐person and within‐person effects, Prati (2025) demonstrated that the relationship between job satisfaction and life satisfaction appears to reflect stable, trait‐like individual differences.

Moreover, drawing causal inferences from observational data becomes even more challenging in the presence of time‐varying predictors and confounders (Hernán and Robins 2020; Robins and Hernán 2008). For instance, according to bottom‐up theory, satisfaction with free time, as a life domain, is expected to influence overall life satisfaction. However, satisfaction with another life domain (e.g., satisfaction with personal relationships) could be a confounder of the causal effect of satisfaction with free time. This is because satisfaction with personal relationships may influence satisfaction with free time and is also likely to be associated with overall life satisfaction due to shared underlying causes. Moreover, both satisfaction with free time and personal relationships change over time. Therefore, the investigation of time‐varying effects in an observational study requires an adjusted set of time‐varying confounders in addition to stable baseline covariates (Hernán and Robins 2020; Robins and Hernán 2008). In such situations, standard regression methods for confounding adjustment are likely to lead to incorrect causal conclusions (Loh et al. 2024). Marginal structural models provide an established solution for pursuing causal inferences in longitudinal studies (Loh et al. 2024). The empirical inconsistencies reported in previous research may be attributed to the “spurious” (or noncausal) associations between life domains and global satisfaction induced by confounders that can vary over time during the course of the study.

### 1.2 Purpose of the Present Study

The main aim of the current study was to test the predictions of both top‐down and bottom‐up perspectives. Based on the bottom‐up perspective (Andrews and Withey 1976; Campbell et al. 1976), the evaluations of various life domains are expected to predict a global evaluation of one's life (Heller et al. 2004). In this study, two analytic approaches were adopted. First, a series of marginal structural models was employed to estimate the effect of satisfaction with each life domain (namely, health, job, relationships, finances, and free time) on global life satisfaction and vice versa while adjusting for the potential time‐varying confounding effects of the other life domains. Second, a continuous time dynamic modeling approach was used to investigate within‐person associations involving both top‐down and bottom‐up processes.

## 2 Method

### 2.1 Procedure and Sample

Data from the Swiss Household Panel study (SHP; SHP Group 2024; Tillmann et al. 2018) were used. The Swiss Household Panel study is a nationwide representative longitudinal survey conducted in Switzerland. The SHP has been running annually since 1999. As the Satisfaction With Life Scale (SWLS; Diener et al. 1985) was used in 2012, 2015, and 2018, the current study used data from 2011 to 2019 (i.e., 1 year prior to the first assessment and 1 year after the last assessment of global life satisfaction). The study complied with ethical standards, and informed consent was obtained from each respondent. The representativeness of the Swiss Household Panel study has been assessed in previous research (Tillmann et al. 2016). Detailed information on the data collection, compliance with ethical standards, sampling methodology, and response rates of SHP is available from Tillmann et al. (2022).

Data from 25,181 participants were used in the current study. The sample consisted of 49% male participants and 51% female participants. Participants' mean age was 47.53 years (SD = 18.15). Their mean years of education (based on ISCED classification) were 11.15 (SD = 5.20). Finally, 86% of participants indicated Swiss as their first nationality (citizenship), while the remaining participants indicated other countries.

### 2.2 Instrument

To measure global life satisfaction, the Satisfaction With Life Scale (SWLS; Diener et al. 1985) was used. The SWLS is a five‐item instrument designed to assess global judgments of satisfaction with one's life. Participants were asked to indicate how satisfied they are with their life using an 11‐point scale that ranges from 0 (*not at all satisfied*) to 10 (*completely satisfied*). An average score was computed such that higher scores indicated greater life satisfaction. Cronbach's alpha ranged from 0.82 to 0.85 across the three measurement occasions.

To measure satisfaction with life domains, all of the domain satisfaction ratings available from the three waves of the SHP (i.e., 2012, 2015, and 2018) were used, except for those that (1) referred only to a specific group of participants (e.g., young people; “satisfaction with the atmosphere with your fellow pupils/students”), (2) had overlapping content (e.g., “satisfaction with free time” and “satisfaction with leisure activities”), or (3) reflected a subdomain of a broader construct (e.g., “satisfaction with the level of interest in tasks,” which is a subdomain of job satisfaction). Specifically, participants were asked to respond to questions about their satisfaction with five domains. The five domain‐specific evaluations included health, income, personal relationships, free time, and job. Ratings ranged from 0 (*not at all satisfied*) to 10 (*completely satisfied*). Such single‐item life satisfaction measures have been shown to be reliable and valid (e.g., Cheung and Lucas 2014; Lucas and Brent Donnellan 2012; Lucas and Donnellan 2007). Participants also responded to questions asking them about their demographics, including age, sex, education, and citizenship.

### 2.3 Analytic Strategy

The current study investigated the predictions of top‐down and bottom‐up theories using two distinct analytic approaches: Hierarchical Bayesian continuous time dynamic modeling and marginal structural model. All analysis scripts are publicly available (https://osf.io/xwmp2/?view_only=7a252a197e1941c9b7dd7ddcc0d831f4).

**FIGURE 1:**
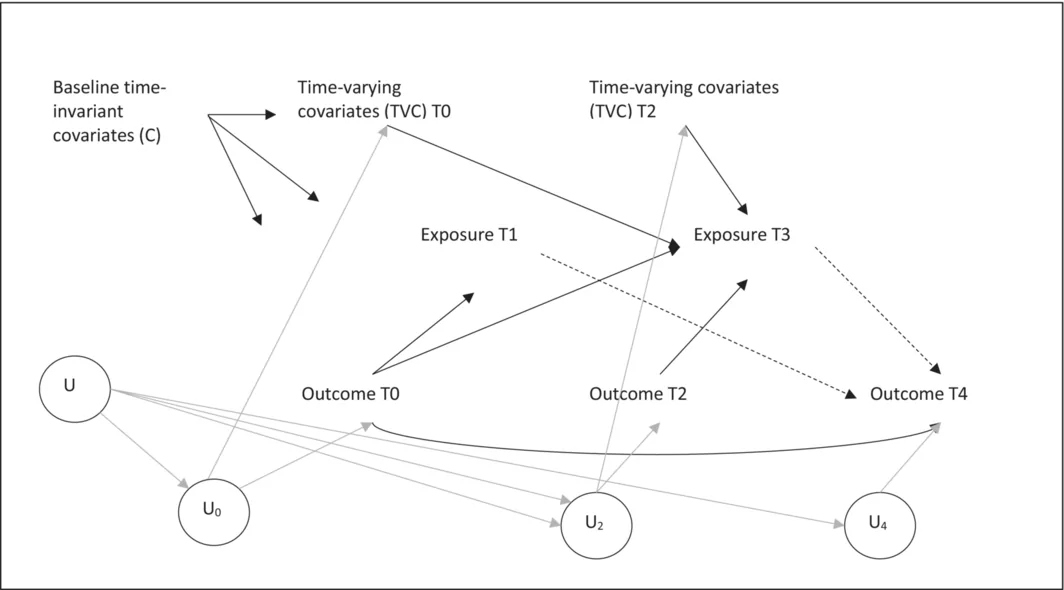
Directed acyclic graph of hypothesized time‐varying effects and confounding in the present study. For visual simplicity, arrows representing the relationships between baseline time‐invariant covariates and observed time‐varying variables are reduced and truncated in the directed acyclic graph. The circular nodes U represents a set of unmeasured variables and the arrows emanating from them are in gray color for visual clarity. Paths corresponding to the effects of the independent variables are drawn as dashed arrows. Baseline time‐invariant covariates were gender, age, education, and citizenship. Time‐varying covariates were all the domain‐specific evaluations except the domain under investigation.

A marginal structural model requires two steps. In the first step, a propensity score model is used to estimate the probability of exposure (i.e., the probability of being exposed to the independent variable), and, for each subject, the inverse probability of exposure weights is calculated. The R package WeightIt (Greifer 2024b) was used to compute the weights from the propensity score model. To perform statistical inference with missing values in the propensity score model, an approach based on a stochastic approximation version of the EM algorithm was used via the R package misaem (Jiang et al. 2020). To assess covariate balance, the R package cobalt (Greifer 2024a) was used. The threshold for covariate balance on correlations was set at 0.1 (Zhu et al. 2015). In the second step, the outcome model is fitted. In the outcome model, the effect of the independent time‐varying variable on the outcome is estimated for the (weighted) pseudo‐population. Baseline covariates were included in the outcome model to make the effect estimate “doubly robust” (a doubly robust estimation is less susceptible to bias arising from model misspecification) and to decrease the bias attributable to residual imbalance. To address missing data in the outcome model, a full information maximum likelihood (FIML) approach via the R package lavaan was used (Rosseel 2012). In the outcome model, the effects of life satisfaction evaluations (either global‐ or domain‐specific) at each time point can be referred to as controlled direct effects. In other words, they are controlled for future exposures (i.e., future levels of the independent variable), and the intermediate process by which the exposure leads to the outcome is not modeled (Mulder et al. 2024). Ninety‐five percent nonparametric percentile bootstrap confidence intervals were obtained using 1000 replications. Figure 1 depicts the directed acyclic graph. In Figure 1, different assessments of the same variable are shown as different nodes. The outcome is the predicted variable, depending on the theoretical perspective. That is, when testing the predictions of top‐down theory, the outcomes are satisfaction with life domains. When testing the predictions of bottom‐up theory, the outcome is global life satisfaction. Exposure is the predictor of interest. When testing the predictions of top‐down theory, the predictor is global life satisfaction. When testing the predictions of bottom‐up theory, the predictors are satisfaction with life domains. Two types of confounders were included in the analyses: time‐varying and time‐invariant covariates (e.g., gender). For simplicity, time‐invariant and time‐varying covariates were denoted collectively as C and TVC, respectively. The arrows represent the direction of influence among the variables. The node U denotes a set of unmeasured variables that affect both the confounder and the outcome. To allow time for the effect of the exposure on the outcome to develop, only the outcome at the last time point is included. Time‐varying covariates were measured 1 year prior to the independent variable. The time lags between the exposure at T1 and at T3 and the outcome at T4 are 4 years and 1 year, respectively. A series of marginal structural models was performed to compute the effects of each independent variable. As time‐invariant covariates, gender, age, education, and citizenship were included in the marginal structural models because previous research has highlighted their role in life satisfaction (e.g., Bartram 2020; Cheung and Chan 2009; Joshanloo and Jovanović 2020; Kirmanoğlu and Başlevent 2014).

Continuous‐time dynamic modeling (Driver and Voelkle 2018, 2021; Hecht and Voelkle 2019) was employed using the R Package ctsem (Driver and Voelkle 2018, 2021). The continuous‐time model is given by the following equation (Hecht and Voelkle 2019):

$$d\theta_{j}\left(t\right)=\left(A\theta_{j}\left(t\right)+b+b_{j}\right)dt+{GdW}_{j}\left(t\right)$$

with

$$Q=GG^{\prime }$$

where the column vector $d\theta_{\mathrm{jp}}$ denotes the manifest responses of person *j* at measurement occasion *p* on each variable; A is the square drift matrix (autoregression matrix in the discrete‐time parameter labels) including the cross‐effects (cross‐lagged effect in the discrete‐time parameter labels) on the off‐diagonals and continuous‐time auto‐effects (autoregressive effect in the discrete‐time parameter labels) on the main diagonal; *Q* represents the symmetric diffusion covariance matrix (process error matrix in the discrete‐time parameter labels) containing the diffusion covariances (process error covariance in the discrete‐time parameter labels) on the off‐diagonals and the diffusion variances (process error variance in the discrete‐time parameter labels) on the main diagonal; b is a column vector with continuous‐time intercepts (corresponding to the discrete‐time intercepts); and *b*_*j*_ is a column vector with individual specific deviations from the continuous‐time intercepts; and the diffusion covariance matrix *Q* = *GG′* represents the covariance matrix of the noise where *G* is the Cholesky factor of the matrix *Q* representing the effect of the noise.

In the R package ctsem, Bayesian fitting using Stan was performed. A Bayesian method was used to deal with missing data (Enders 2025). The uncertainty of the missing data Bayesian method provides a natural approach to modeling. The posterior 95% Bayesian credible intervals (CIs) were calculated. Figure S2 shows a LaTeX representation of the model. In the hierarchical continuous time model, there were 12 free model parameters referring to global satisfaction (GS) and domain satisfaction (DS):

The drift matrix (A) with continuous‐time auto‐effects and cross‐effects:

$$A=\left[\begin{array}{cc} a_{\mathrm{GS}} & a_{\mathrm{DS}\to \mathrm{GS}}\\ a_{\mathrm{GA}\to \mathrm{DS}} & a_{\mathrm{DS}} \end{array}\right]$$

The diffusion covariance matrix (Q):

$$Q=\left[\begin{array}{cc} \sigma_{\mathrm{GS}}^{2} & \sigma_{\mathrm{DS}↔\mathrm{GS}}\\ \sigma_{\mathrm{GA}↔\mathrm{DS}} & \sigma_{\mathrm{DS}}^{2} \end{array}\right]$$

The continuous‐time intercepts (b):

$$b=\left[\begin{array}{c} b_{\mathrm{GS}}\\ b_{\mathrm{DS}} \end{array}\right]$$

The covariance matrix of continuous‐time intercepts:

$$\sum b=\left[\begin{array}{cc} \sigma_{\mathrm{GS}}^{2} & \sigma_{\mathrm{DS}↔\mathrm{GS}}\\ \sigma_{\mathrm{GA}↔\mathrm{DS}} & \sigma_{\mathrm{DS}}^{2} \end{array}\right]$$

In this model, as the continuous‐time intercepts were set to individually varying, stable individual differences (unobserved heterogeneity) were accounted for. Therefore, the effects of between‐ and within‐person variation were disentangled. In addition, the Λ matrix (LAMBA option) was a 2 × 2 matrix with the value 1 on the diagonal, meaning that each latent variable was directly reflected in the observed variable. The measurement error terms (MANIFESTVAR option) were defined as zero because of the limited number of time points available. Finally, the measurement intercept (τ; MANIFESTMEANS option) was set to zero for identification (continuous‐time intercepts were estimated).

## 3 Results

### 3.1 Missing Data Analysis

Table S1 (supplemental material) reports the number of missing values for each primary study variable and the correlations (Spearman coefficients) with the amount of missing data per participant. According to the effect size guidelines of Gignac and Szodorai (2016), the magnitudes of the correlation coefficients were very small (i.e., |*r*| < 0.1) for all variables. This suggests that the amount of missing data per participant was not meaningfully related to any of the primary study variables. Table S2 presents the number of missing data patterns and the frequency of cases within each pattern. Twelve missing data patterns were found. On average, participants had 1.99 missing values.

### 3.2 Marginal Structural Models

A series of marginal structural models was employed to test the predictions of the top‐down and bottom‐up theories. Before estimating each marginal structural model, balance was assessed. Achieving balance is necessary to justify ignorability on the observed covariates, thereby allowing for valid conclusions after effect estimation. With continuous exposures (i.e., independent variables), the Pearson correlation between each covariate and exposure provides information about covariate balance. Although correlations should ideally be close to zero, employing a threshold for balance statistics helps determine whether adequate covariate balance has been achieved. Zhu et al. (2015) recommend that absolute correlations should be no greater than 0.1. The adjusted correlations between each covariate and the exposure variable never exceeded the recommended threshold of 0.1 (Zhu et al. 2015). Table 1 displays the results from a series of five marginal structural models predicting each domain‐specific evaluation from global life satisfaction. With the exception of the effects of Time 1 global life satisfaction on Time 4 satisfaction with health, financial situation, and free time, the predictions of the top‐down theory were supported. Table 2 reports the results from a series of five marginal structural models predicting global life satisfaction from each domain‐specific evaluation. The predictions of the bottom‐up theory were supported. The only exception was a nonsignificant effect of Time 3 satisfaction with free time on Time 4 global life satisfaction.

**TABLE 1:** Results from a series of five marginal structural models predicting each domain‐specific evaluation from global life satisfaction (SWLS).

Variable	Estimate	95% CI	*p*
Time 4 satisfaction with health			
Time 1 global life satisfaction	−0.02	−0.13, 0.08	0.680
Time 3 global life satisfaction	0.25	0.12, 0.38	< 0.001
Time 4 job satisfaction			
Time 1 global life satisfaction	0.12	0.01, 0.23	0.041
Time 3 global life satisfaction	0.21	0.09, 0.32	0.001
Time 4 satisfaction with personal relationships			
Time 1 global life satisfaction	0.11	0.02, 0.19	0.012
Time 3 global life satisfaction	0.21	0.12, 0.31	< 0.001
Time 4 satisfaction with financial situation			
Time 1 global life satisfaction	0.07	−0.03, 0.18	0.212
Time 3 global life satisfaction	0.38	0.26, 0.50	< 0.001
Time 4 satisfaction with free time			
Time 1 global life satisfaction	0.10	−0.004, 0.21	0.066
Time 3 global life satisfaction	0.14	0.03, 0.26	0.020

*Note:* Estimates are unstandardized. Baseline time‐invariant covariates were gender, age, education, and migration status. Time‐varying covariates were all the domain‐specific evaluations except the domain under investigation (i.e., the outcome).

Abbreviation: CI, confidence interval.

**TABLE 2:** Results from a series of five marginal structural models of associations between each domain‐specific evaluation and subsequent global life satisfaction (SWLS).

Variable	Time 4 global life satisfaction (SWLS)		
	Estimate	95% CI	*p*
Time 1 satisfaction with health	0.09	0.06, 0.12	< 0.001
Time 3 satisfaction with health	0.07	0.04, 0.10	< 0.001
Time 1 job satisfaction	0.12	0.08, 0.15	< 0.001
Time 3 job satisfaction	0.05	0.02, 0.09	0.004
Time 1 satisfaction with personal relationships	0.12	0.09, 0.15	< 0.001
Time 3 satisfaction with personal relationships	0.10	0.06, 0.13	< 0.001
Time 1 satisfaction with financial situation	0.08	0.05, 0.10	< 0.001
Time 3 satisfaction with financial situation	0.09	0.06, 0.11	< 0.001
Time 1 satisfaction with free time	0.03	0.01, 0.05	0.004
Time 3 satisfaction with free time	0.02	−0.004, 0.04	0.081

*Note:* Estimates are unstandardized. Baseline time‐invariant covariates were gender, age, education, and migration status. Time‐varying covariates were all the domain‐specific evaluations except the domain under investigation (i.e., the exposure).

Abbreviation: CI, confidence interval.

### 3.3 Dynamics of the Top‐Down and Bottom‐Up Processes

Hierarchical Bayesian continuous time dynamic modeling was used to estimate the auto‐effects and cross‐effects, derived from the drift matrix, characterizing the temporal dynamics of the top‐down and bottom‐up processes. The top‐down process was represented by scores on the SWLS, while the bottom‐up process was represented by the mean scores across all domains of life satisfaction. Cross‐effects represent the effect of one process on the other. The cross‐effects of the bottom‐up process on the top‐down process, estimate = 0.70, 95% CI [0.23, 1.13], and of the top‐down process on the bottom‐up process, estimate = 0.69, 95% CI [0.44, 0.95], were positive and significant. An independent shock of magnitude 1.00 to the bottom‐up process predicted a hypothetical future increase—according to the fitted model—in the top‐down process and vice versa. To facilitate the interpretation of the cross‐effects, Figure S1 (supplemental material) illustrates the dynamic regression coefficients implied by the model over a six‐year interval.

### 3.4 Robustness Analysis

**FIGURE 2:**
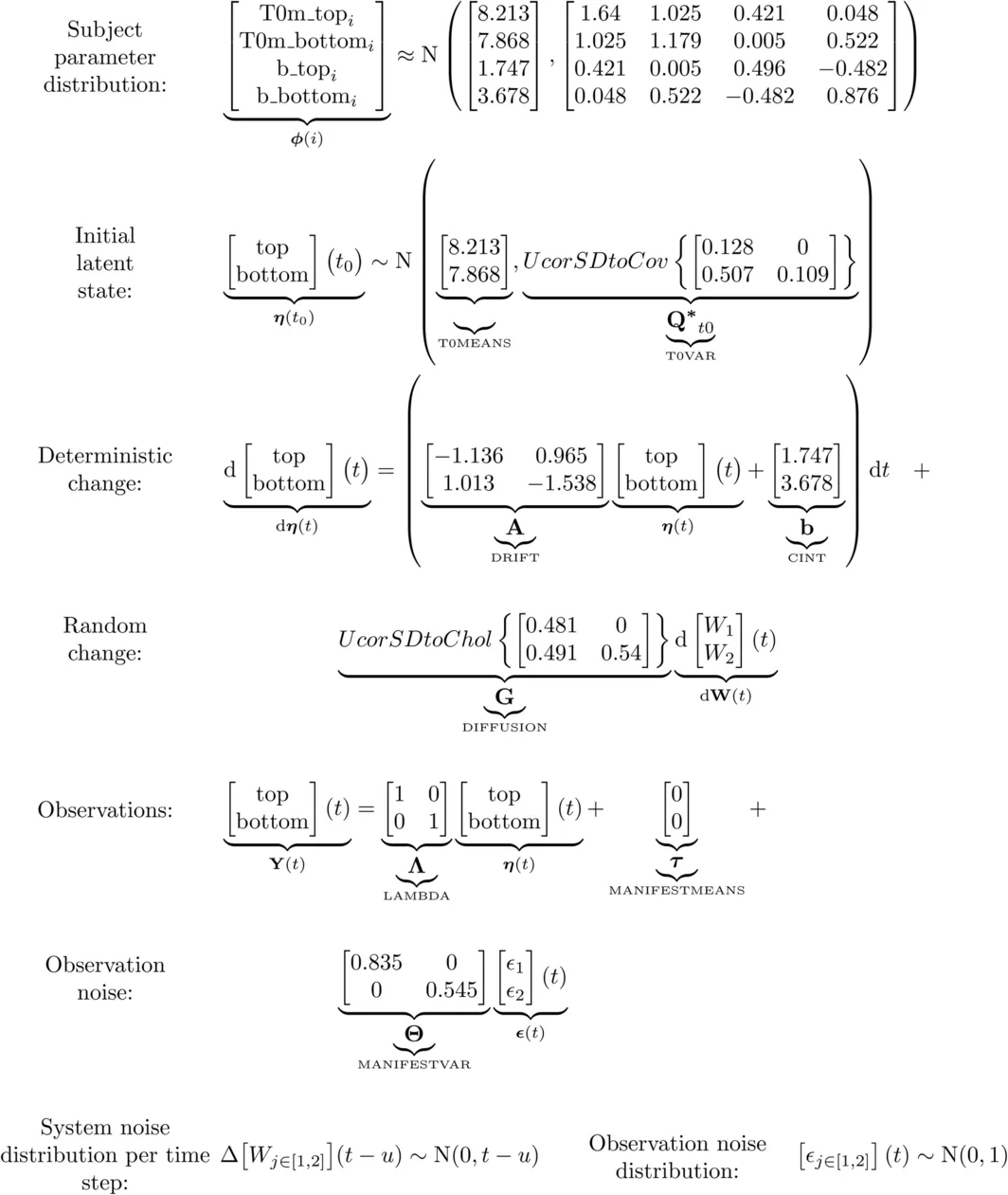
LaTeX representation of the parameter estimates of the hierarchical continuous time model (robustness analysis). *UcorSDtoChol* converts lower tri matrix of standard deviations and unconstrained correlations to Cholesky factor, *UcorSDtoCov* = transposed cross product of *UcorSDtoChol*, to give covariance (see Driver and Voelkle (2018), 11). Individual specific notation (subscript i) only shown for subject parameter distribution—pop. means shown elsewhere. Linearized approximation of subject parameter distribution shown.

One anonymous Reviewer requested an extension of the analysis to include a greater number of waves using all available waves that included both single‐item life satisfaction (substituting for the Satisfaction With Life Scale) and domain‐specific ratings. In the Swiss Household Panel study, the five domain ratings were examined in the annual surveys from Wave 6 to Wave 22 (the latest available wave at the time of the study). Accordingly, the robustness analysis employed hierarchical Bayesian continuous time dynamic modeling across 15 waves. Another Reviewer asked to address the measurement error and system noise correlation aspects in this model (Driver 2023) by including a larger number of waves. Therefore, in this analysis, the assumption of no measurement error was relaxed, and consideration of the process noise correlation was provided. Figure S3 presents a LaTeX‐formatted representation of the model. The cross‐effects of the top‐down process on the bottom‐up process, estimate = 0.96, 95% CI [0.95, 0.97], and of the bottom‐up process on the top‐down process, estimate = 1.01, 95% CI [1.01, 1.02], were positive and significant. The correlation in system noise was 0.49, 95% CI [0.47, 0.51]. Figure 2 reports all parameter estimates using a LaTeX formatted representation of the equations. Figures S4 and S5 display the dynamic regression coefficients considering not only the deterministic component but also the stochastic component of the system as suggested by Driver (2023). Taken together, the findings from this robustness analysis corroborate the main results.

One anonymous Reviewer requested that the appropriateness of FIML as a method for handling missing data be established by investigating whether the findings differed without such an approach. Tables S3 and S4 report the findings without employing the FIML approach. The results did not differ substantially.

## 4 Discussion

The aim of the current study was to investigate the predictions of top‐down and bottom‐up perspectives. The findings of the current study indicate that both top‐down and bottom‐up processes are relevant to understanding life satisfaction. Therefore, the findings support early theoretical formulations identifying bottom‐up factors contributing to life satisfaction. Satisfaction with life domains, such as health, income, relationships, free time, and job, may fulfill basic human needs. According to the theory of Wilson (1967), the fulfillment of these needs determines life satisfaction. Although satisfaction with different life circumstances predicted global life satisfaction, people in distinct developmental life stages (Buecker et al. 2023) and from different cultures (Suh and Koo 2008) may prioritize and weigh satisfaction with life circumstances differently. The findings of the current study also provide support for the top‐down perspective. Global life evaluations predict a wide range of domain‐specific satisfaction, shaping how people perceive, experience, and cope with events in multiple domains of their lives. The role of global life satisfaction as well as the weights attributed to multiple life domains are subject to considerable variation between and within (over the life course) individuals and across cultures (e.g., Cantor and Sanderson 1999; Comolli et al. 2024; Oishi 2006).

The novelty of the current study lies in providing evidence about how the effects of top‐down and bottom‐up theories may unfold over time. For instance, the bottom‐up perspective suggests that individuals mentally combine their evaluations of various life domains to form global life evaluations, whereas the top‐down perspective posits the reverse. The findings of this longitudinal study cannot be attributed solely to mental operations. Rather, the findings can be interpreted as an examination of how the impact of global and domain‐specific evaluations unfolds over time. The results of the hierarchical Bayesian continuous‐time dynamic modeling revealed that when participants experience a change in global evaluations, they also tend to experience a subsequent change in domain‐specific evaluations, and vice versa. These reciprocal effects tend to diminish over time. In this analysis, stable individual differences (unobserved heterogeneity) were controlled for, allowing within‐person dynamics to be investigated (Hamaker 2023). The results of the marginal structural models revealed the population‐level average potential outcomes that would have been observed in terms of hypothetical manipulations of previous levels of the predictors. A marginal structural modeling approach allows for straightforward estimation of the effects over time (Loh et al. 2024; Robins and Hernán 2008). The results of the hierarchical Bayesian continuous‐time dynamic modeling and the marginal structural models were consistent.

The results of the current study are in line with those of previous research documenting support for both the bottom‐up and top‐down models of life satisfaction (e.g., Heller et al. 2004; Lance et al. 1989; Malvaso and Kang 2022). It is likely that prior findings favoring one theoretical perspective over the other (e.g., Busseri and Mise 2020; Headey et al. 1991; Leonardi et al. 2005) might be due to confounding bias (Hernán and Robins 2020; Loh et al. 2024) or the conflation of within‐person and between‐person variability (Curran and Bauer 2010; Hamaker 2023).

## 5 Implications for Theory and Practice

Taken together, the findings of this study can be interpreted through the lens of feedback loop theory. This perspective contrasts with the view that top‐down and bottom‐up processes represent competing theories. Indeed, theory and evidence support the view that bottom‐up and top‐down theories can be combined in a single integrated model (e.g., Malvaso and Kang 2022). However, it is worth noting that the lower limit of the confidence intervals was sometimes too close to zero to be considered robust. For instance, the lower bound of the confidence interval for the effect of Time 3 job satisfaction on Time 4 global life satisfaction approached zero, suggesting that an increase in job satisfaction may not consistently lead to increases in global life satisfaction. Moreover, in line with recent research, the relationship between job satisfaction and life satisfaction tends to be reciprocal when statistically significant (Prati 2025). The relative scope, criticality, and centrality of life domains might influence the strength of the reciprocal relationship between global and domain‐specific evaluations (Lance et al. 1989).

According to the top‐down perspective, the global evaluations of one's life are the product of stable dispositional factors (e.g., basic personality traits, genetic factors) that are thought to determine how people perceive, interpret, and respond to life events and circumstances. In addition, when facing negative life circumstances such as a pandemic, most people may display a trajectory of life satisfaction indicative of resilience (e.g., Prati and Mancini 2023, 2024). While interventions on these dispositional factors may be difficult or even unattainable, practitioners can focus on the promotion of positive domain‐specific evaluations, which are more dependent on life events and circumstances (Busseri and Mise 2020). For instance, the trajectories for satisfaction with one's finances and satisfaction with one's health may be impacted by changes in income and health status, respectively (Bunda and Busseri 2019). In addition to interventions aimed at addressing life circumstances, positive psychology interventions (e.g., Kwok et al. 2016) and worksite wellness interventions (e.g., Berkland et al. 2017) may prove useful.

### 5.1 Limitations

While the marginal structural models accounted for the effects of time‐varying predictors and confounding (Hernán and Robins 2020; Robins and Hernán 2008), causal inference relies on several assumptions, such as the sequential ignorability assumption (also termed no unmeasured confounding). Because an unmeasured confounder could always theoretically exist, it is very difficult—if not impossible—to draw definitive conclusions. Moreover, the findings of the study are limited to participants from only one European country. Further research is needed to evaluate the generalizability of the findings across different countries and cultural contexts. Additional factors influencing top‐down and bottom‐up processes—such as genetic predispositions, life events, and personal circumstances—were not investigated in the current study. These additional factors may be particularly relevant for predicting global and domain‐specific evaluations. Future research could extend the present work by investigating additional determinants of top‐down and bottom‐up processes. Furthermore, although the current work included a range of domain‐specific evaluations, the range of additional life domains that may influence—and be influenced by—global life satisfaction is potentially infinite. The inclusion of other domain‐specific evaluations should be considered in future research to ensure that the findings are broadly applicable.

## 6 Conclusion

The findings of the current study suggest that top‐down and bottom‐up processes predict each other through a feedback mechanism. Over time, a feedback loop between top‐down and bottom‐up processes may develop, and their reciprocal predictive effects can perpetuate both high and low levels of life satisfaction.

## Author Contributions

Gabriele Prati is currently associate professor (fixed‐term) at the Department of Psychology of the University of Bologna. He has extensive research and academic as well as professional experience. His research interests focus on mental health, job satisfaction, happiness, attitudes, well‐being, and life satisfaction.

## Funding

The author has nothing to report.

## Disclosure

*Artificial Intelligence*: No artificial intelligence assisted technologies were used in this research or the creation of this article. *Materials*: All study materials are publicly available (https://forscenter.ch/projects/swiss‐household‐panel/documentation/). *Analysis Scripts*: Analysis scripts for main analyses are publicly available (https://osf.io/xwmp2/?view_only=7a252a197e1941c9b7dd7ddcc0d831f4).

## Ethics Statement

As the Swiss Household Panel data are publicly available, and confidentiality is protected by identity masking, this study was exempt from institutional review board ethical assessment. The Swiss Household Panel obtained informed consent from all participants.

## Conflicts of Interest

The author declares no conflicts of interest.

## Supporting information


**Data S1:** jopy70039‐sup‐0001‐supinfo.docx.

## Data Availability

This study has been realized using data collected by the Swiss Household Panel (SHP), which is based at the Swiss Centre of Expertise in the Social Sciences FORS. The project is supported by the Swiss National Science Foundation. The author cannot share the data collected by the Swiss Household Panel because it is protected by data protection policy. Notwithstanding, all study materials are available via the SWISSUbase catalogue after creating an account and signing a license agreement (https://doi.org/10.48573/n4ns‐5830).
