# Supplementary material for: The Relationship Between Global and Domain‐Specific Evaluations of Life Satisfaction: A Feedback Loop Theory
Source: J Pers. 2025 Dec 12;94(5):783–93. doi: 10.1111/jopy.70039 (PMC13540322; doi:10.1111/jopy.70039)
Supplement: Supplementary file 1 [file JOPY-94-783-s001.docx]

**Table S1**

*Number of Missing Data for Each Variable and Correlation (Spearman Coefficients) with the Amount of Missing Data Per Participant*

| Variable | *Number of missing data* | *r_s_* |
| --- | --- | --- |
| Domain-specific life satisfaction 2012 | *6495* | *-.01* |
| Domain-specific life satisfaction 2015 | *2753* | *.04* |
| Domain-specific life satisfaction 2018 | *4591* | *.03* |
| Global satisfaction (SWLS) 2012 | *6494* | *.03* |
| Global satisfaction (SWLS) 2015 | *2746* | *.05* |
| Global satisfaction (SWLS) 2018 | *4590* | *.07* |

**Table S2**

*Number Of Missing Data Patterns and the Number of Cases in Each of the Patterns*

| Number of cases | Domain-specific life satisfaction 2012 | Domain-specific life satisfaction 2015 | Domain-specific life satisfaction 2018 | Global satisfaction (SWLS) 2012 | Global satisfaction (SWLS) 2015 | Global satisfaction (SWLS) 2018 | Number of missingness |
| --- | --- | --- | --- | --- | --- | --- | --- |
| 4688 | 1 | 1 | 1 | 1 | 1 | 1 | 0 |
| 3264 | 0 | 1 | 1 | 0 | 1 | 1 | 2 |
| 1 | 0 | 1 | 0 | 0 | 1 | 1 | 3 |
| 1149 | 1 | 1 | 0 | 1 | 1 | 0 | 2 |
| 1 | 0 | 1 | 0 | 1 | 1 | 0 | 3 |
| 2082 | 0 | 1 | 0 | 0 | 1 | 0 | 4 |
| 2 | 0 | 0 | 1 | 0 | 1 | 1 | 3 |
| 6 | 0 | 0 | 0 | 0 | 1 | 0 | 5 |
| 1 | 0 | 1 | 1 | 0 | 0 | 1 | 3 |
| 255 | 1 | 0 | 1 | 1 | 0 | 1 | 2 |
| 1138 | 0 | 0 | 1 | 0 | 0 | 1 | 4 |
| 1352 | 1 | 0 | 0 | 1 | 0 | 0 | 4 |

*Note.* Each row corresponds to a missing data pattern (1=observed, 0=missing).

**Table S3**

*Results from a Series of Five Marginal Structural Models Predicting Each Domain‐Specific Evaluation from Global Satisfaction (SWLS)*

| Variable | Estimate | 95% CI |
| --- | --- | --- |
| Time 4 Satisfaction with health | | |
| Time 1 Global satisfaction | -0.02 | .762 |
| Time 3 Global satisfaction | 0.22 | .002 |
| Time 4 Job Satisfaction | | |
| Time 1 Global satisfaction | 0.06 | .196 |
| Time 3 Global satisfaction | 0.24 | *p* <.001 |
| Time 4 Satisfaction with personal relationships | | |
| Time 1 Global satisfaction | 0.12 | .011 |
| Time 3 Global satisfaction | 0.19 | *p* <.001 |
| Time 4 Satisfaction with financial situation | | |
| Time 1 Global satisfaction | 0.07 | .229 |
| Time 3 Global satisfaction | 0.38 | *p* <.001 |
| Time 4 Satisfaction with free time | | |
| Time 1 Global satisfaction | 0.05 | .510 |
| Time 3 Global satisfaction | 0.10 | .157 |

*Note.* Estimates are unstandardized. CI =confidence interval. Baseline time-invariant covariates were gender, age, education, and migration status. Time-varying covariates were all the domain‐specific evaluations except the domain under investigation (i.e., the outcome).

**Table S4**

*Results from a Series of Five Marginal Structural Models of Associations Between Each Domain‐Specific Evaluation and Subsequent Global satisfaction (SWLS)*

| Variable | Time 4 Global satisfaction (SWLS) | |
| --- | --- | --- |
|  | Estimate | *p* |
| Time 1 Satisfaction with health | 0.05 | .004 |
| Time 3 Satisfaction with health | 0.07 | <.001 |
| Time 1 Job Satisfaction | 0.05 | .016 |
| Time 3 Job Satisfaction | 0.07 | .001 |
| Time 1 Satisfaction with personal relationships | 0.07 | .001 |
| Time 3 Satisfaction with personal relationships | 0.09 | <.001 |
| Time 1 Satisfaction with financial situation | 0.05 | <.001 |
| Time 3 Satisfaction with financial situation | 0.07 | <.001 |
| Time 1 Satisfaction with free time | 0.03 | .020 |
| Time 3 Satisfaction with free time | 0.02 | .060 |

*Note.* Estimates are unstandardized. CI =confidence interval. Baseline time-invariant covariates were gender, age, education, and migration status. Time-varying covariates were all the domain‐specific evaluations except the domain under investigation (i.e., the exposure).

**Figure S1**

*Top-down and bottom-up processes: Discrete-time cross-lagged standardized parameters for varying time intervals based on an independent 1-unit change on each process*

**Figure S2**

*LaTeX Formatted Representation of the Equations of the Hierarchical Continuous Time Model*

**Figure S3**

*LaTeX Formatted Representation of the Equations of the Hierarchical Continuous Time Model (Robustness Analysis)*

**Figure S4**

Robustness Analysis: Temporal Dynamics Effects, With an Independent Shock of Magnitude 1.00 to Each Construct

**Figure S5**

Robustness Analysis: Temporal Dynamics Effects, With a Correlated Shock of Magnitude 1.00 to Each Construct
